# Stereoscopic vs. monoscopic photographs on optic disc evaluation and glaucoma diagnosis among general ophthalmologists: A cloud-based real-world multicenter study

**DOI:** 10.3389/fmed.2022.990611

**Published:** 2022-10-13

**Authors:** Jingyuan Yang, Yi Qu, Jianchun Zhao, Jingwu Cong, Zixi Sun, Yunfeng Du, Gang Yang, Dayong Ding, Youxin Chen, Gangwei Cheng

**Affiliations:** ^1^Department of Ophthalmology, Peking Union Medical College Hospital, Peking Union Medical College, Chinese Academy of Medical Sciences, Beijing, China; ^2^Key Laboratory of Ocular Fundus Diseases, Chinese Academy of Medical Sciences, Beijing, China; ^3^Department of Ophthalmology, Peking University Third Hospital, Haidian District, Beijing, China; ^4^Visionary Intelligence Ltd, Beijing, China; ^5^Key Lab of DEKE, Renmin University of China, Beijing, China

**Keywords:** diagnosis, glaucoma, optic disc, photograph, stereoscopic

## Abstract

**Purpose:**

To investigate whether stereoscopic vs. monoscopic viewing condition influences the evaluation of optic disc photographs for morphologic features and glaucoma likelihood in a general ophthalmologist population from multicenters on a cloud-based platform.

**Methods:**

A cross-sectional study of 519 pairs of stereoscopic and monoscopic photographs of optic discs with adequate quality were selected and presented using a cloud-based platform. A total of 21 general ophthalmologists from 14 centers assessed 15 morphologic features based on 5R's rules and estimated glaucoma likelihood for each assigned photograph. There were 93 pairs of stereoscopic and monoscopic photographs evaluated by a panel of glaucoma specialists and set as ground truth. The main outcome measures were the agreement between estimates and ground truth and the inter-grader agreements.

**Results:**

There were good agreements between ground truth and both monoscopic and stereoscopic estimates (stereo κ 0.532 and mono κ 0.494). There was also a substantial intra-grader agreement between monoscopic and stereoscopic evaluation of glaucoma likelihood (κ 0.636). In eyes with probable glaucoma, the accuracy of the stereo method was greater than that of the mono method (stereo 0.238 vs. mono 0.118) When compared with ground truth, stereoscopic photographs had a better agreement for disc size (stereo κ 0.447 vs. mono κ 0.183), disc color (stereo κ 0.612 vs. mono κ 0.549), neuroretinal rim shape (stereo κ 0.356 vs. mono κ 0.274) on the whole. The stereoscopic method also had a better inter-grade agreement for disc size, disc color, neuroretinal rim shape, and glaucoma likelihood (stereo κ 0.402 vs. mono κ 0.359) on the whole.

**Conclusions:**

In the evaluation of optic disc photographs for morphologic features and glaucoma likelihood, the stereoscopic method showed superiority compared to the monoscopic method for general ophthalmologists. The stereoscopic method is more likely to identify glaucomatous eyes which need medical intervention.

## Introduction

Accurate and reproducible assessment of the optic disc and adjacent retinal structures using images of the optic disc has a key role in the evaluation of the condition of the optic disc and in the diagnosis and management of glaucoma (1–3). Taking photographs is currently the main method for optic disc documentation, and both monoscopic and stereoscopic methods have been widely used in clinical practice. Monoscopic optic disc photographs have relative advantages in convenience and cost, while stereoscopic ones provide more topographic information, which has been one of the gold standards for detecting glaucomatous optic disc (4, 5).

However, previous studies showed mixed results when comparing stereoscopic and monoscopic photographs in evaluating optic disc conditions, and the performance of the stereoscopic method has not been evaluated among general ophthalmologists. Several studies found that stereoscopic photographs had a similar inter-grader agreement among glaucoma specialists or experts to that of their monoscopic counterparts (6, 7), while some studies reported significant variability in non-stereo and stereo photographs among glaucoma specialists when evaluating optic disc (8–10). The differences between stereo and mono methods might come from the fact that stereoscopic photos provide a better understanding of the three-dimensional structure of the optic disc theoretically. In order to find a better method for accurate analysis of optic disc and optimum management of glaucomatous patients, a comparison between the stereoscopic method and its monoscopic counterpart is necessary.

For a good consistency of evaluations for the optic disc photographs, a standardized method to obtain stereoscopic images is necessary when comparing various photographic methods as well as taking photos in clinical practice. However, previous methodological studies mostly used sequential stereoscopic images (6–10), rather than simultaneous stereoscopic images, of which the sequential technique usually requires changing the position and angle of the camera manually to produce a horizontal offset and thus introduces bias due to lack of standardization. In contrast, a camera that allows a simultaneous record of side-by-side images with a synchronous fixed angle and the same condition of exposure could provide standardized images and theoretically more consistent with the real appearance of the optic disc.

Moreover, the assessing procedure of optic discs is usually subjective, which highly relies on extensive experience to achieve high diagnostic precision (11). However, the precision may not be applicable to general healthcare providers in real-world clinical practice (9). Considering that a great proportion of patients who visit clinics for glaucoma screening were examined by ophthalmologists with experience that might not equal to that of glaucoma experts, systematic and strategic observation of every feature of the optic disc on photographs by general ophthalmologists is necessary.

This study employs a cloud-based standardized assessment system for simultaneous stereoscopic photos that were taken in a real-world clinical setting. This study was designed to compare accuracy and agreement for a series of optic disc parameters and glaucoma likelihood of stereoscopic optic disc photographs with those of monoscopic photographs by general ophthalmologists in a real-world multicenter clinical setting.

## Materials and methods

Approval from Peking Union Medical College Hospital's institutional review board was obtained for this project (approval number S-K2061), and the research was conducted in accordance with the Declaration of Helsinki. All data collected from the institutions were analyzed anonymously. As this study involved an anonymous medical record review with no more than minimal risk to participants, it met all requirements for a waiver of informed consent per institutional policy.

### Data collection

Six hundred pairs of monoscopic and stereoscopic photographs from 600 eyes were recruited consecutively from the Department of Ophthalmology, Peking Union Medical College Hospital. These clinic-based photographs were captured in our clinical practice and selected for further evaluation. None had a history of coexisting ocular diseases, a history of intraocular surgery, or systemic diseases with possible ocular involvement. Photographs of inadequate quality were excluded because they might not exhibit the differences between monoscopic and stereoscopic photographs. Exclusive criteria of image quality included poor illumination of the disc, poor quality image, lens opacity, poor stereoimage, and optic discs of anomalous configuration (those which were totally tilted, congenital abnormal, or high myopic). A total of 519 pairs of images were determined to be suitable for further evaluation by a masked glaucoma specialist.

Each pair of photographs included a monoscopic photograph at a 45-degree field of view and stereoscopic photographs at a 20 × 27 degree field of view. The photographs were taken using a Kowa nonmydriatic retinal camera WX 3D (Kowa, Tokyo, Japan) (examples in Supplementary Figure 1). Photographs were saved as TIFF files (monoscopic) and JPEG files (stereoscopic). Images were uploaded to an interactive platform (https://anno.vistel.cn, one example webpage on this website is shown in Supplementary Figure 1) for further annotating, diagnosing, and grading.

### Optic disc assessment

Five R's Rules were followed when assessing optic discs to establish a standardized system for comprehensive evaluation of morphologic features of optic discs without omission (12). The morphologic features included disc size, disc color, disc shape, disc contour, neuroretinal rim shape, ISNT rule consistency, cup-to-disc ratio, retinal nerve fiber layer, beta zone, hemorrhage, and small vessels (Table 1). Furthermore, we set several quantitative thresholds for metrics instead of subjective evaluation. For the range of retinal nerve fiber layer defect (RNFLD), quantitative analysis was performed in the superotemporal (10 to 12 clock h for right eyes, and 12 to 2 clock h for left eyes) and inferotemporal (6 to 8 clock h for right eyes, and 4 to 6 clock h for left eyes) quadrants, while the range of hemorrhages was evaluated all circle around the disc. For the range of RNFLD, a non-overlapping range of more than 1 clock h in each evaluated quadrant between two graders was regarded as inconsistency. Similarly, for the range of hemorrhages, inconsistency was defined as a non-overlapping range of more than 0.5 clock h.

**Table 1:** Evaluation scale of morphologic characteristics and glaucoma diagnosis based on the Five R's Rules and 4-subcategory glaucoma likelihood classification.

**Morphologic characteristics based on Five R's Rule**	**Scale**
R1	
Disc size	Normal | Abnormal (including small, large, and not sure)
Disc color	Normal | Abnormal (including rosy, pallor, pale, and not sure)
Disc shape	Normal | Abnormal/Tilt
Disc contour	Clear | Unclear (including blurring, and not sure)
R2	
Neuroretinal rim shape	Normal | Abnormal (including suspected and abnormal)
Abnormal rim shape	Localized thinning | Notching | Diffuse thinning
ISNT rule consistency	Yes | No
CDR	Quantitative measurements, including vertical CDR, and area CDR, by identifying manually
R3	
Retinal nerve fiber layer (RNFL)	Normal | Abnormal
Abnormal RNFL	Quantitative measurements for RNFL defect range
R4	
Zone beta	Presence | Absence
Contour of zone beta	Clear | Unclear
R5	
Retinal and optic disc hemorrhages	Presence | Absence
Presence of hemorrhages	Quantitative measurements for hemorrhagic range
Small vessels	Normal | Abnormal
Abnormal small vessels	Narrowing | Tortuosity or distortion | Bridging
Diagnosis	
Glaucoma likelihood	None | Possible | Probable | Definite

CDR, cup-to-disc ratio; ISNT, I for inferior, S for superior, N for nasal, T for temporal.

Glaucoma likelihood was classified into four subcategories based on optic disc appearance: definite, probable, suspect, and none glaucoma (Table 1) (4, 13, 14). This detailed classification system could help distinguish subtle differences between monoscopic and stereoscopic photos, which might contribute to corresponding therapy according to gradings of risks or severities of glaucoma.

In order to set a gold standard for training and assessment, 93 pairs of stereoscopic and monoscopic photographs from 93 eyes were selected randomly. These photos were evaluated based on 5R's rule and 4-scale glauoma likelihood classification. These results were further discussed and assessed by an expert panel of five glaucoma professors *via* video meeting. In cases of disagreement, the leading glaucoma specialist (GW.C) made the final decision. These estimates assessed by glaucoma experts were set as ground truth.

**Figure 1:**
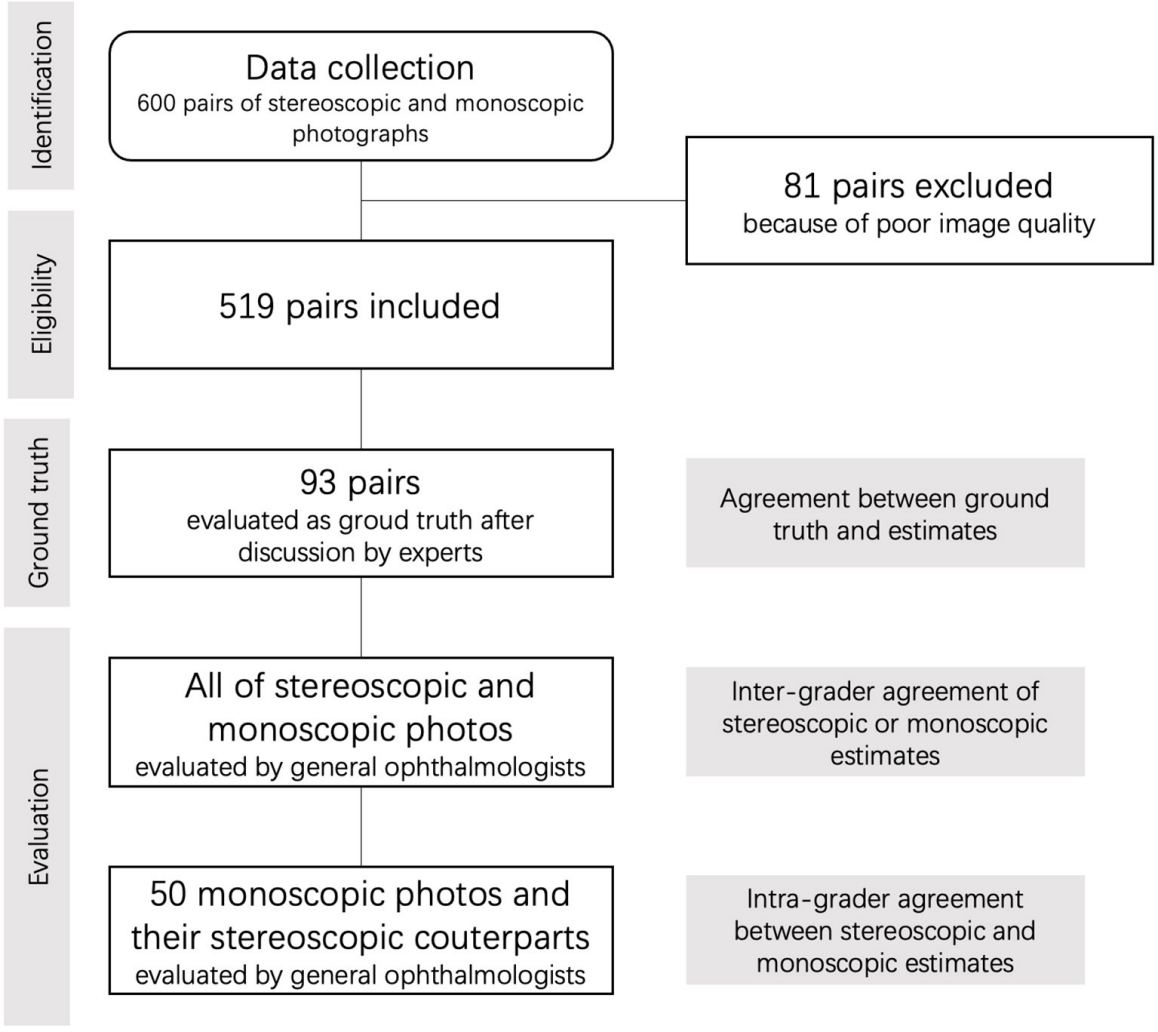
Flow chart of the current study.

There were 21 volunteer national certificated general ophthalmologists of various seniority of clinical practice, from 14 various hospitals, who participated in analyzing and grading the whole photograph set. Of these, 18 ophthalmologists had worked for more than 3 years, and at least seven ophthalmologists were fellows or attendings while the other ophthalmologists worked as residents. All of them were fully trained with 5 R's Rules and 4-scale glaucoma likelihood classification in an offline workshop until they could estimate the stereoscopic and monoscopic photos based on the same standard in tests. They could discuss with a glaucoma specialist (GW.C) if they had any question about the process of grading in training. They were grouped randomly into four groups. There were four rounds of annotation throughout the process, with about 75 pairs of photographs per round (Supplementary Table 1). Mandatory evaluation of the photographs from the abovementioned 93 eyes was required, and their estimates were used to evaluate their agreement with the ground truth. The inter-grader agreement of stereoscopic and monoscopic photos was evaluated, respectively. For each grader, about 50 monoscopic photographs whose corresponding stereoscopic ones have been evaluated by themselves were selected randomly and estimated for intra-grader agreement (Figure 1). Each photo was evaluated by at least two graders. In order to wash out the memory effect, the monoscopic and stereoscopic photos were dispatched to ophthalmologists in various batches over at least a 1-month interval. In order to provide a standardized environment during evaluation, all of these graders were assigned uniform stereo glasses, and the brightness, contrast, size, and resolution of images were adjusted automatically on the cloud-based platform. Glasses for correcting refractive error were recommended, but the ambient lightning were not standardized forcibly because the environment in which ophthalmologists view the photos in clinical practice were not same all across the world.

### Statistics

The levels of agreement for each morphologic characteristic were calculated. Agreement for nominal variables was calculated using weighted kappa (κ). The kappa is a numerical value that ranges from −1 (complete disagreement) to +1 (total agreement). For the continuous variables, the agreement was assessed using the intraclass correlation coefficient (ICC). The degree of agreement was classified according to the value of kappa or ICC as follows: slight (0–0.2), fair (0.2–0.4), moderate (0.4–0.6), substantial (0.6–0.8), and almost perfect (0.8–1) (15). Differences between various kappa or ICC values were considered statistically if the mean value for one viewing method lay outside two standard deviations of the mean value for the other viewing method (7, 16). When further and direct comparisons were available, paired *t*-test was performed.

Intra-grader agreement was computed by comparing the evaluating results of each morphologic characteristic using stereoscopic photographs and their matching monoscopic counterparts. Inter-grader agreements of the stereoscopic method and monoscopic method were calculated by comparing each grader's answers to variables, respectively. Agreement for each feature between estimates and ground truth was also calculated.

All statistical analysis, other than kappa statistics, was performed using SPSS 25.0 (IBM, Chicago, IL, USA). Kappa statistics were performed using a custom algorithm with Python.

## Results

**Figure 2:**
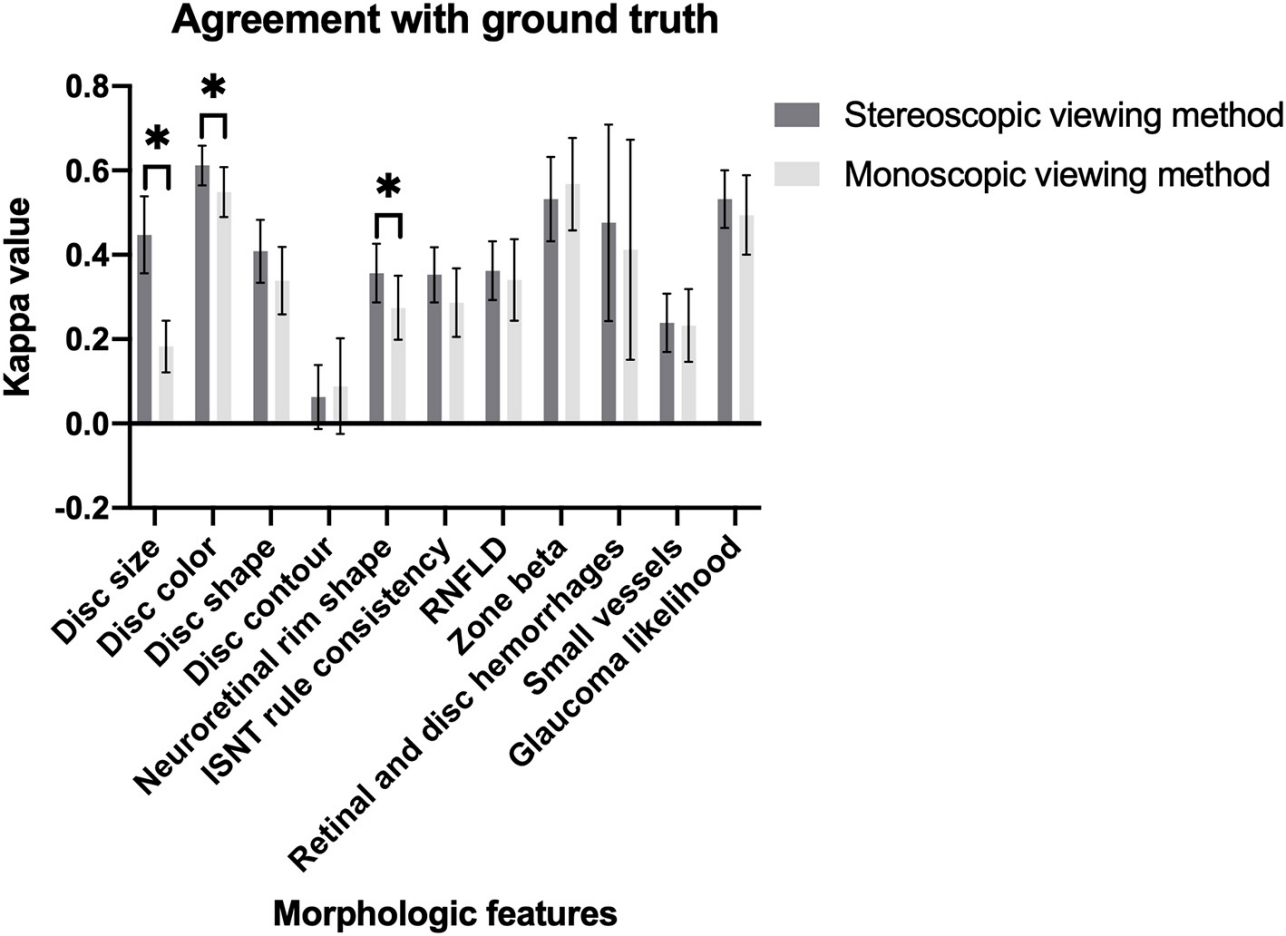
Magnitude of agreement between two viewing methods and ground truth for morphologic features of optic disc and glaucoma likelihood (*P < 0.05).

**Figure 3:**
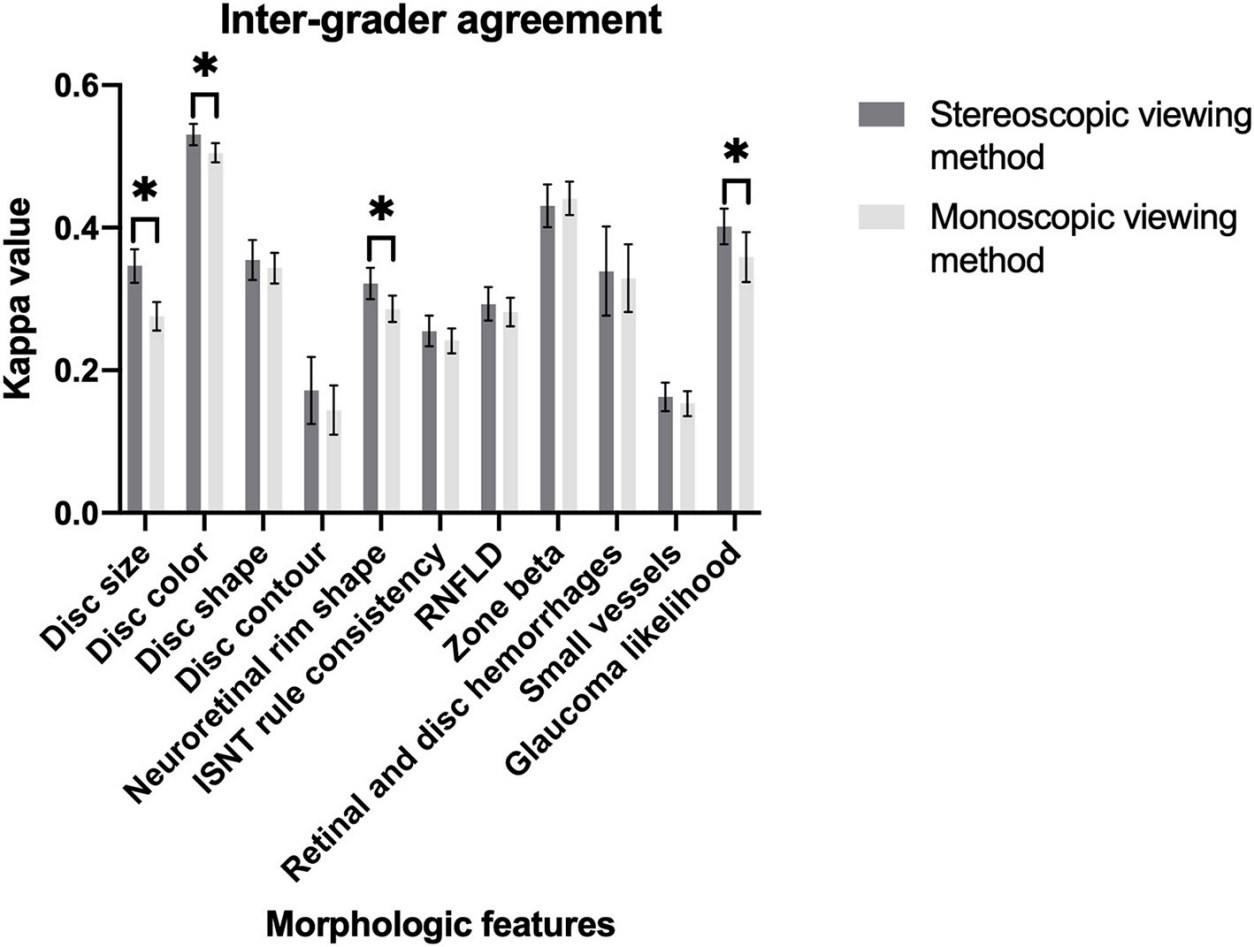
Magnitude of inter-grader agreement of different viewing methods for morphologic features of optic disc and glaucoma likelihood (*P < 0.05).

A total of 600 simultaneous stereoscopic photographs and their monoscopic counterparts were used for the evaluation of optic discs and glaucoma likelihood. Although both stereoscopic and monoscopic photos showed good agreement with the ground truth of glaucoma likelihood, the stereoscopic photos had better accuracy than monoscopic did for probable glaucoma (stereo κ 0.238 vs. mono κ 0.118). The stereoscopic method had better agreement with the ground truth than the monoscopic did when evaluating disc size, disc color, and neuroretinal rim shape. On the contrary, the monoscopic method misled graders more easily on disc size in probable glaucoma with significance, and RNFLD in definite and probable glaucoma with borderline significance. The overall levels of agreements for each morphologic feature and glaucoma likelihood between ground truth and viewing method were shown in Figure 2. The overall levels of inter-grader agreement of various viewing methods for each morphologic feature and glaucoma likelihood were shown in Figure 3. Detailed values were listed in Supplementary Tables 2–13.

### R1: Scleral ring for optic disc and its size

The agreement between ground truth and stereoscopic estimates was superior or comparable to that between ground truth and monoscopic estimates for disc size [stereo κ 0.447, confidence interval (CI) 0.356–0.539 vs. mono κ 0.183, CI 0.121–0.244], disc color (stereo κ 0.612, CI 0.565–0.659 vs. mono κ 0.549, CI 0.490–0.608), disc shape (stereo κ 0.409, CI 0.334–0.483, and mono κ 0.339, CI 0.259–0.419), and disc contour (stereo κ 0.063, CI −0.013–0.139 and mono κ 0.088, CI −0.025–0.202) (Supplementary Table 2).

The inter-grader agreement using the stereoscopic method was also significantly greater than or similar to that using the monoscopic method for disc size (stereo κ 0.347, CI 0.323–0.370 vs. mono κ 0.276, CI 0.256–0.296), disc color (stereo κ 0.531, CI 0.516–0.546 vs. mono κ 0.505, CI 0.492–0.519), disc shape (stereo κ 0.355, CI 0.327–0.383 vs. mono κ 0.344, CI 0.322–0.365), and disc contour (stereo κ 0.172, CI 0.125–0.219 vs. mono κ 0.144, CI 0.110–0.179), as well as in each glaucoma likelihood subcategories, except for disc size and disc color in eyes with probable glaucoma (Supplementary Tables 3–5).

These two viewing methods had a substantial intra-grader agreement for disc color (κ 0.619) and disc shape (κ 0.568), and fair intra-grader agreement for disc size (κ 0.252) and disc contour (κ 0.278).

### R2: Optic disc rim

For neuroretinal rim shape, the agreement between ground truth and stereoscopic estimates was greater than that between ground truth and monoscopic estimates (stereo κ 0.356, CI 0.287–0.426 vs. mono κ 0.274, CI 0.199–0.350), and the inter-grader agreement using the stereo method was better than that using mono method (stereo κ 0.322, CI 0.300–0.344 vs. mono κ 0.286, CI 0.268–0.305) (Supplementary Table 6).

The agreements of ISNT rule consistency between ground truth and both monoscopic and stereoscopic estimates were fair (stereo κ 0.353, CI 0.287–0.418, and mono κ 0.286, CI 0.405–0.368). The levels of inter-grader agreement for stereoscopic and monoscopic assessments showed no significant differences (stereo κ 0.255, CI 0.234–0.277 vs. mono κ 0.242, CI 0.224–0.259). Compared with the mono method, the stereo method showed better inter-grader agreement in probable glaucoma and unsatisfying inter-grader agreement in suspect glaucoma (Supplementary Table 7).

These viewing methods had moderate intra-agreement for neuroretinal rim shape (κ 0.487) and ISNT rule consistency (κ 0.427).

When assessing vertical CDR, stereoscopic assessments showed slightly greater value than monoscopic assessments (stereo 0.690 ± 0.111 vs. mono 0.684 ± 0.106, *P* 0.003). In subcategory evaluation, the vertical CDRs in stereoscopic photos of suspect glaucoma were slightly greater than those in monoscopic photos (stereo 0.686 ± 0.101 vs. mono 0.677 ± 0.099, *P* 0.011), while other subcategories of glaucoma likelihood showed no significant differences of vertical CDR between monoscopic and stereoscopic estimates. The area CDR in stereoscopic photos was also greater than that in monoscopic photos (stereo 0.449 ± 0.133 vs. mono 0.443 ± 0.125, *P* 0.001), and this phenomenon was noticed in definite and suspect glaucoma (Supplementary Table 8).

### R3: Retinal nerve fiber layer

When assessing RNFLD, the agreements between ground truth and both monoscopic and stereoscopic photos were both moderate and similar (stereo κ 0.36, CI 0.29–0.43 vs. mono κ 0.34, CI 0.24–0.44), and the levels of inter-grader agreement for monoscopic and stereoscopic estimates were similar as well (stereo κ 0.29, CI 0.27–0.32 vs. mono κ 0.28, CI 0.26–0.30). But the inter-grader agreement of the mono method was greater than that of the stereo method in eyes with definite glaucoma and probable glaucoma. For the eyes with RNFLD, graders had a slightly higher inter-grader agreement in stereoscopic estimates than that in monoscopic estimates when detecting RNFLD (stereo 51.6 vs. mono 47.3%). The κ value of intra-grader agreement between these two methods was 0.538 (Supplementary Table 9).

### R4: Region of parapapillary atrophy

The level of agreement between monoscopic estimates and ground truth for the beta zone was moderate (κ 0.568, CI 0.458–0.677), which was similar to the level between stereoscopic estimates and ground truth (κ 0.532, CI 0.432–0.632). The inter-grader agreements for the beta zone in stereoscopic and monoscopic estimates, on the whole, were relatively fair, and no significant difference between these viewings was found (stereo κ 0.431, CI 0.401–0.461 vs. mono κ 0.441, CI 0.418–0.465). Except in eyes with definite and suspect glaucoma, monoscopic photos showed better inter-grader agreements (Supplementary Table 10).

Both stereoscopic viewing and monoscopic viewing showed no significant agreement with ground truth (stereo κ −0.005, CI −0.055–0.044 vs. mono κ 0.109, CI −0.027–0.246), and these viewing methods showed no significant difference. Although stereoscopic and monoscopic methods had similar inter-grader agreements on the whole (stereo κ 0.234, CI 0.176–0.291 vs. mono κ 0.214, CI 0.170–0.257), but the stereoscopic method had better inter-grader agreements in eyes with definite, suspect and none glaucoma (Supplementary Table 11).

The levels of intra-grader agreement for the beta zone and its contour were substantial (κ 0.623 and 0.455, respectively).

### R5: Retinal and optic disc hemorrhages and small vessels

For retinal and optic disc hemorrhages, stereoscopic viewing and monoscopic viewing had similar agreements with ground truth (stereo κ 0.476, CI 0.243–0.709 vs. mono κ 0.412, CI 0.151–0.673). They also had similar inter-grader agreements on the whole (stereo κ 0.339, CI 0.277–0.402 vs. mono κ 0.329, CI 0.282–0.377) (Supplementary Table 12).

When evaluating small vessels, the levels of agreements between ground truth and stereo estimates and between ground truth and mono estimates showed no significant difference (stereo κ 0.239, CI 0.170–0.308 vs. mono κ 0.232, CI 0.146–0.319). Although stereoscopic and monoscopic methods had similar inter-grader agreements on the whole (stereo κ 0.163, CI 0.143–0.183 vs. mono κ 0.154, CI 0.136–0.171), but the monoscopic method had better inter-grader agreements in eyes without glaucoma (Supplementary Table 13).

### Glaucoma likelihood

**Figure 4:**
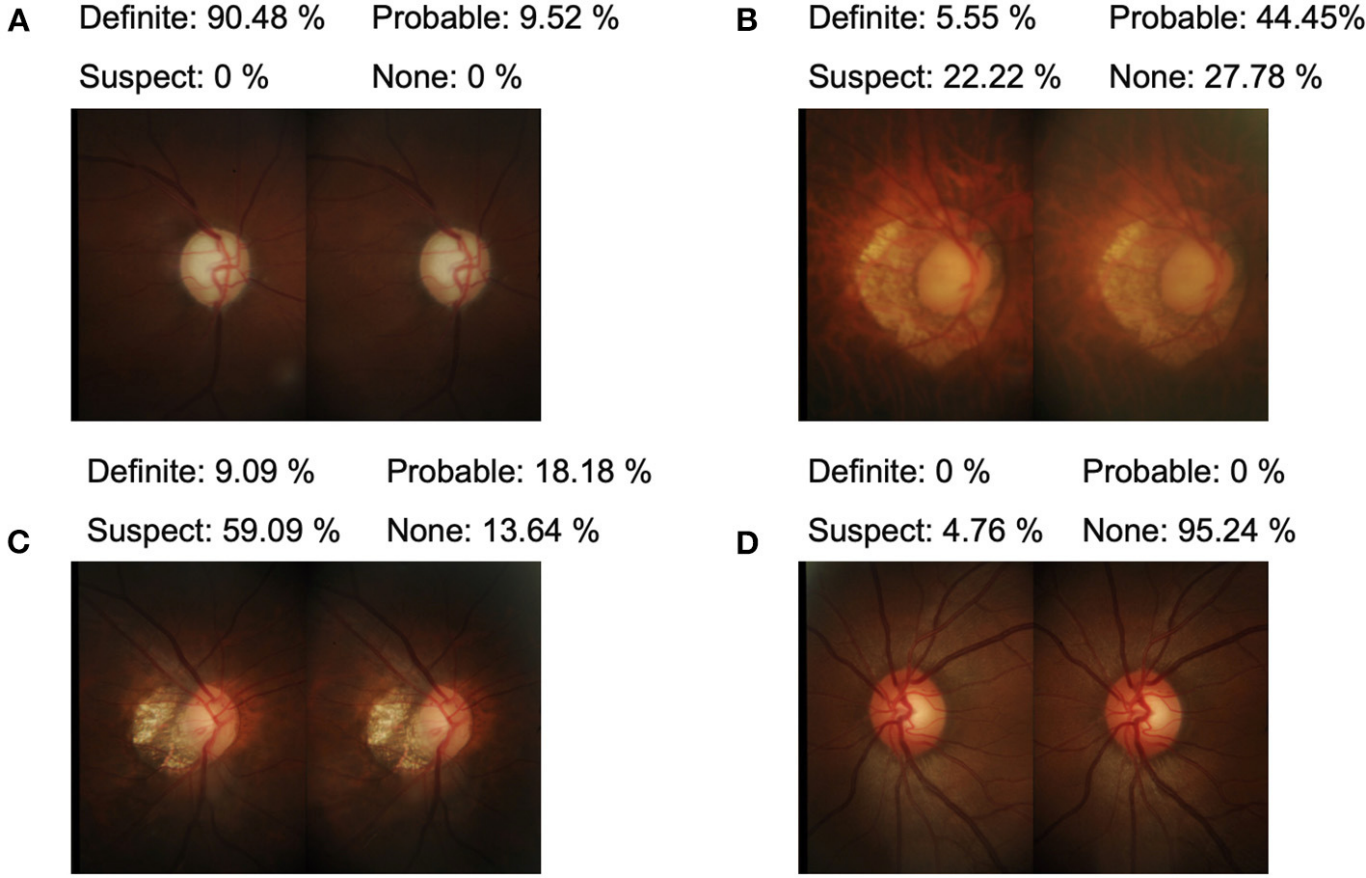
Examples of representative optic disc photographs of various glaucoma likelihood **(A)**, definite glaucoma; **(B)**, possible glaucoma; **(C)**, probable glaucoma; **(D)**, none glaucoma. The distribution of gradings is shown above corresponding images.

There were good agreements between ground truth and both monoscopic and stereoscopic estimates (stereo κ 0.532, CI 0.464–0.600 and mono κ 0.494, CI 0.400–0.589) (Table 2, examples are shown in Figure 4). In eyes with probable glaucoma, the accuracy of the stereo method was greater than that of the mono method (stereo κ 0.238, CI 0.101–0.375 vs. mono κ 0.118, CI 0.005–0.230) (Supplementary Table 14). There was a substantial intra-grader agreement between the monoscopic and stereoscopic evaluation of glaucoma likelihood (κ 0.636, CI 0.551–0.720).

**Table 2:** The levels of agreement for glaucoma likelihood.

**Viewing method**	**Agreement with ground** **truth (kappa)**	**Inter-grader** **agreement (kappa)**	**Agreement between two viewing** **viewing methods (kappa)**
Stereoscopic	0.532 (0.464–0.600)	0.402^*^ (0.377–0.427)	0.636 (0.377–0.427)
Monoscopic	0.494 (0.400–0.589)	0.359^*^ (0.324–0.394)	

* Statistically significant.

The stereoscopic method had better inter-grader agreement than the monoscopic method did (stereo κ 0.402, CI 0.377–0.427 vs. mono κ 0.359, CI 0.324–0.394), especially in eyes with definite glaucoma, but not in eyes with probable glaucoma and suspect glaucoma.

## Discussion

The present study has shown that for general ophthalmologists in the real world, there were some significant differences in evaluating morphologic characteristics of the optic disc and estimating glaucoma likelihood when using stereoscopic photographs of the optic disc compared to monoscopic photographs. The results of the current study demonstrated that assessment of glaucoma likelihood with the stereoscopic method showed superior performance than the monoscopic method, especially in distinguishing eyes with probable glaucoma. The stereoscopic method had superiority in identifying glaucomatous eyes which need medical interventions. There was substantial agreement in glaucoma likelihood assessment between stereoscopic and monoscopic methods (κ 0.636). However, the stereoscopic estimates had a greater inter-grader agreement on the whole, and better accuracy in eyes with probable glaucoma. When compared with ground truth, stereoscopic photographs had a better agreement for disc size, disc color, and neuroretinal rim shape, on the whole. The stereoscopic method also presented a better inter-grade agreement for disc size, disc color, neuroretinal rim shape, and glaucoma likelihood on the whole. On the contrary, the monoscopic method showed no overall superiority for any estimated features.

We used a 4-subcategory classification of glaucoma likelihood in the present study. The grading level is incremented according to the clinical likelihood of glaucoma and various management, in which none glaucoma only needs regular examinations, suspect glaucoma needs intensive monitoring, probable glaucoma needs treatment without setting target intraocular pressure (IOP), and definite glaucoma needs treatment with target IOP setting. Compared with the 2-subcategory classification of discriminating only glaucomatous and nonglaucomatous eyes, the more detailed classification with four subcategories helps identify the extent of risks for each individual and provides personalized management in clinical practice.

Criteria for this classification of glaucoma likelihood was based on the characteristics of the optic nerve head. Five R's Rules assist ophthalmologists to observe optic discs comprehensively in a standardized workflow, and reduce the risk of missing information (13, 14). This detailed classification enhances the ability to detect differences between stereo and mono methods as well as probably increases the difficulty of accurate grading for general ophthalmologists. In the current study, the inter-grader agreement for 4-subcategory glaucoma likelihood reached 0.4 under stereoscopic conditions and 0.35 under monoscopic conditions. In another study evaluating 4-subcategory glaucoma likelihood by 21 glaucoma specialists from multiple international centers, Kong et al. reported the κ value of inter-observer agreement reached 0.63 (17). Although expert consensus assessment demonstrated higher performance in assessment, our results may reflect optic disc assessment in clinical practice, which reaches a moderate and acceptable level. However, the inter-grader agreement might reduce when using a more refined classification system. Varma et al. reported that inter-observer agreement for 2-subcategory glaucoma diagnosis was 0.50 using the stereo method, which was assessed by six experts (10). Reus et al. reported that inter-observer agreement for 2-subcategory glaucoma diagnosis using stereo photos reached 0.72 for general ophthalmologists, and 0.45 for residents (18). Moreover, we also investigated the diagnostic accuracy, which reaches an acceptable level (mean κ 0.532) when using stereoscopic photos. Therefore, determining glaucoma likelihood with a 4-subcategory classification system after evaluating optic discs with 5R's Rules is feasible in clinical practice.

Theoretically, stereoscopic photographs provide a better understanding of the three-dimensional structure of the optic disc (7), but we noticed that some differences in estimates between stereoscopic and monoscopic methods need further explanation. For example, stereoscopic viewing provided a volumetric perspective for assessing the optic disc, which enables more precise estimates of the size, color, and shape of optic discs, and neuroretinal rim in this study. Moreover, when assessing ISNT rule consistency, stereoscopic viewing might help identify rim alterations, and especially in eyes with a high risk of glaucoma, the rim changes were more easily to be noticed in stereoscopic photos, especially for eyes with probable glaucoma. The values of CDR in stereoscopic photos were usually greater than those in monoscopic photos, and the rim widths in stereoscopic photos were usually less (9, 10). Therefore, the stereo method had a better inter-grader agreement in eyes with probable glaucoma. Furthermore, when evaluating RNFLD in eyes with definite and probable glaucoma, although inter-grader agreements were greater using the mono method, the agreements with ground truth were relatively less using the mono method, which indicated that the monoscopic method might lead to false classifications of glaucoma more easily. Similarly, although the monoscopic method showed better inter-grader agreement for disc size, disc color in eyes with probable, and for ISNT rule consistency in eyes with suspect glaucoma, they did not exceed stereo counterpart on accuracy, and even had worse agreement with ground truth. Therefore, the stereoscopic method is helpful to provide an objective evaluation. Besides, we noticed that monoscopic methods had a better inter-grader agreement for small vascular abnormalities in nonglaucoma eyes. Although this result did not influence the diagnosis of glaucoma, excessive information from stereoscopic photographs might interfere with the judgement of graders. In contrast, previous studies of comparison between monoscopic and stereoscopic methods did not evaluate the morphological features of optic discs and glaucomatous possibility comprehensively and did not evaluate the stereoscopic methods among general ophthalmologists in the real world (Supplementary Table 15).

The design of the current study applied a series of methods to standardize the evaluation process and enhance its reliability and persuasion. First of all, developing a standardized and comprehensive grading system by applying 5 R's Rules for optic discs, a detailed classification for glaucoma likelihood, and a simultaneous stereo camera can help overcome variability in the process of subjective clinical evaluation (12, 17, 19). Moreover, the number of graders and the number of evaluated photos were greater than those of previous studies. In the current study the 4-subcategory classification of glaucoma likelihood was evaluated by 21 trained general ophthalmologists, while previous studies investigated the 4-subcategory classification of glaucoma likelihood by glaucoma experts or only 2-subcategory classification (10, 17, 18, 20). Considering that level of expertise has been shown to affect stereoscopic photography grading (21), the study could reflect optic nerve head assessment in clinical practice. Furthermore, we used clinic-based photos rather than community-based photos. We excluded a large number of photos of normal optic discs. The relatively complicated conditions of optic discs and glaucoma likelihood in the present study were similar to the clinical practice in the busy clinical setting. Therefore, the methods in the current study could be reproduced in real-world clinical practice.

Considering that stereoscopic photographs provided more detailed and realistic details, these photos could be used to train residents and general ophthalmologists to achieve consistent ability and the same evaluation results, which benefits not only image reviewing but also management of evaluation results. We also assumed that artificial intelligence models trained with stereoscopic photos might be able to provide results that are closer to the truth than those trained with monoscopic counterparts.

The strengths of this study include the relatively large number of annotated photographs and representative graders, its prospective randomized design due to the application of a cloud-based platform, and the same viewing conditions as real-world clinical settings. Therefore, considering that a great proportion of patients who visit clinics for glaucoma screening were examined by ophthalmologists with experience that might not equal that of glaucoma experts, we investigated the value of a comprehensive estimated method on stereoscopic photographs of the optic disc in real-world clinical practice. However, we still have several potential limitations. First, we did not compare ophthalmologists with less experience and expert assessment. As stereo photos could provide topographic information which enables graders to evaluate with a stereoscopic view, graders with less experience might benefit more than experts who may be able to draw reliable clinical judgments using only mono cues. On one hand, the value of experience in evaluating optic discs and estimating glaucoma likelihood was not part of our purpose. On the other hand, the conclusions of our study should not be extended to all levels of ophthalmologists. Second, due to the application of a cloud-based platform, we allowed graders to review and change their previous annotations, which introduced a risk of recall of a previously seen photograph when we evaluate the intra-grader agreement by using stereo and mono photos of the same optic discs. We used several methods to minimize this risk, for example, decreasing the number of photos used for evaluating intra-grader agreement, and setting a washout period. However, we still cannot eliminate the bias because of recall. Third, because of the limited levels of graders' training and expertise, it is inevitable that some judgments during evaluation might lack sufficient reasons and experienced estimates (20), and the repeatability of estimation with photographs of optic disc needs further investigation. Fourth, we did not calculate the single grader's intra-grader agreement of stereoscopic photos or monoscopic photos, because graders are allowed to review and revise their previous grading and annotations on the cloud-based platform, which was similar to the process of reviewing clinical information in clinical practice. Therefore, considering that once the same photo was given twice or more times the graders can evaluate photos based on previous grading and annotations by reviewing previous evaluations, we could not provide the results of this kind of intra-grader agreement in this study. Fifth, due to the intrinsic weakness of calculating kappa value, the kappa value could be amazingly low when one category in a binary variable counts almost all. For example, the accuracy of judging disc contour reaches more than 0.95, but the kappa value for agreement with ground truth was lower than 0.1.

In summary, our analysis showed that general ophthalmologists assessed optic discs with a better inter-grader agreement and diagnostic accuracy for glaucoma likelihood on the whole. The stereoscopic method had superiority in identifying glaucomatous eyes which need medical interventions. The monoscopic method showed no overall superiority for any estimated features in the present study. Stereoscopic optic disc photography is recommended for general ophthalmologists in the clinical evaluation of glaucomatous optic disc damage, and their routine use in real-world clinical settings might compensate for the lack of expertise and experience.

## Data availability statement

The raw data supporting the conclusions of this article will be made available by the authors, without undue reservation.

## Ethics statement

The studies involving human participants were reviewed and approved by Peking Union Medical College Hospital Institutional Review Board. The Ethics Committee waived the requirement of written informed consent for participation. Written informed consent was obtained from the individual(s) for the publication of any potentially identifiable images or data included in this article.

## Author contributions

JY: design, definition of intellectual content, data analysis, manuscript preparation, and manuscript editing. YQ: data acquisition, manuscript preparation, and manuscript review. JZ: data acquisition, definition of intellectual content, data analysis, manuscript preparation, and manuscript editing. JC: data acquisition, data analysis, manuscript preparation, and manuscript review. ZS: data acquisition and manuscript review. YD: data acquisition, data analysis, and manuscript review. GY: design and manuscript review. DD: concepts, design, and manuscript review. YC: concepts and manuscript review. GC: concepts, design, data analysis, manuscript preparation, and manuscript editing. All authors contributed to the article and approved the submitted version.

## Conflict of interest

Authors JZ, JC, YD, and DD were employed by Visionary Intelligence Ltd. The remaining authors declare that the research was conducted in the absence of any commercial or financial relationships that could be construed as a potential conflict of interest.

## Publisher's note

All claims expressed in this article are solely those of the authors and do not necessarily represent those of their affiliated organizations, or those of the publisher, the editors and the reviewers. Any product that may be evaluated in this article, or claim that may be made by its manufacturer, is not guaranteed or endorsed by the publisher.

## Supplementary material

Supplementary Figure 1 Examples of stereoscopic and monoscopic photographs from three glaucomatous eyes. A pair of suitable stereo glasses were strongly suggested when viewing the stereoscopic photographs.

Supplementary Table 1 The number of pairs of stereoscopic and monoscopic photographs evaluated by graders.

Supplementary Table 2 The levels of agreement for disc size. CI, confidence interval. ^*^Statistically significant.

Supplementary Table 3 The levels of agreement for disc color. CI, confidence interval. ^*^Statistically significant.

Supplementary Table 4 The levels of agreement for disc shape. CI, confidence interval. ^*^Statistically significant.

Supplementary Table 5 The levels of agreement for disc contour. CI, confidence interval.

Supplementary Table 6 The levels of agreement for neuroretinal rim shape. CI, confidence interval. ^*^Statistically significant.

Supplementary Table 7 The levels of agreement for ISNT rule consistency. CI, confidence interval; ISNT, I for inferior, S for superior, N for nasal, T for temporal. ^*^Statistically significant.

Supplementary Table 8 The levels of agreement for CDR and comparison of different viewing methods. CDR, cup-to-disc ratio; CI, confidence interval; ICC, intraclass correlation coefficient.

Supplementary Table 9 The levels of agreement for RNFLD. CI, confidence interval; RNFLD, retinal nerve fiber layer defect. ^*^Statistically significant.

Supplementary Table 10 The levels of agreement for beta zone. CI, confidence interval. ^*^Statistically significant.

Supplementary Table 11 The levels of agreement for contour of beta zone. CI, confidence interval. ^*^Statistically significant.

Supplementary Table 12 The levels of agreement for retinal and optic disc hemorrhages. CI, confidence interval.

Supplementary Table 13 The levels of agreement for small vessels. CI, confidence interval. ^*^Statistically significant.

Supplementary Table 14 Inter-grader agreement and accuracy of each subcategory of glaucoma likelihood. CI, confidence interval. ^*^Statistically significant.

Supplementary Table 15 Previous studies of comparison between monoscopic and stereoscopic optic disc photos.

## Abbreviations

CDR: cup-to-disc ratio
CI: confidence interval
ICC: intraclass correlation coefficient
ISNT: I for inferior, S for superior, N for nasal, T for temporal
RNFLD: retinal nerve fiber layer defect.
