# Supplementary figures and images for: Stereoscopic vs. monoscopic photographs on optic disc evaluation and glaucoma diagnosis among general ophthalmologists: A cloud-based real-world multicenter study
Source: Front Med (Lausanne). 2022 Oct 13;9:990611. doi: 10.3389/fmed.2022.990611 (PMC9612717; doi:10.3389/fmed.2022.990611)

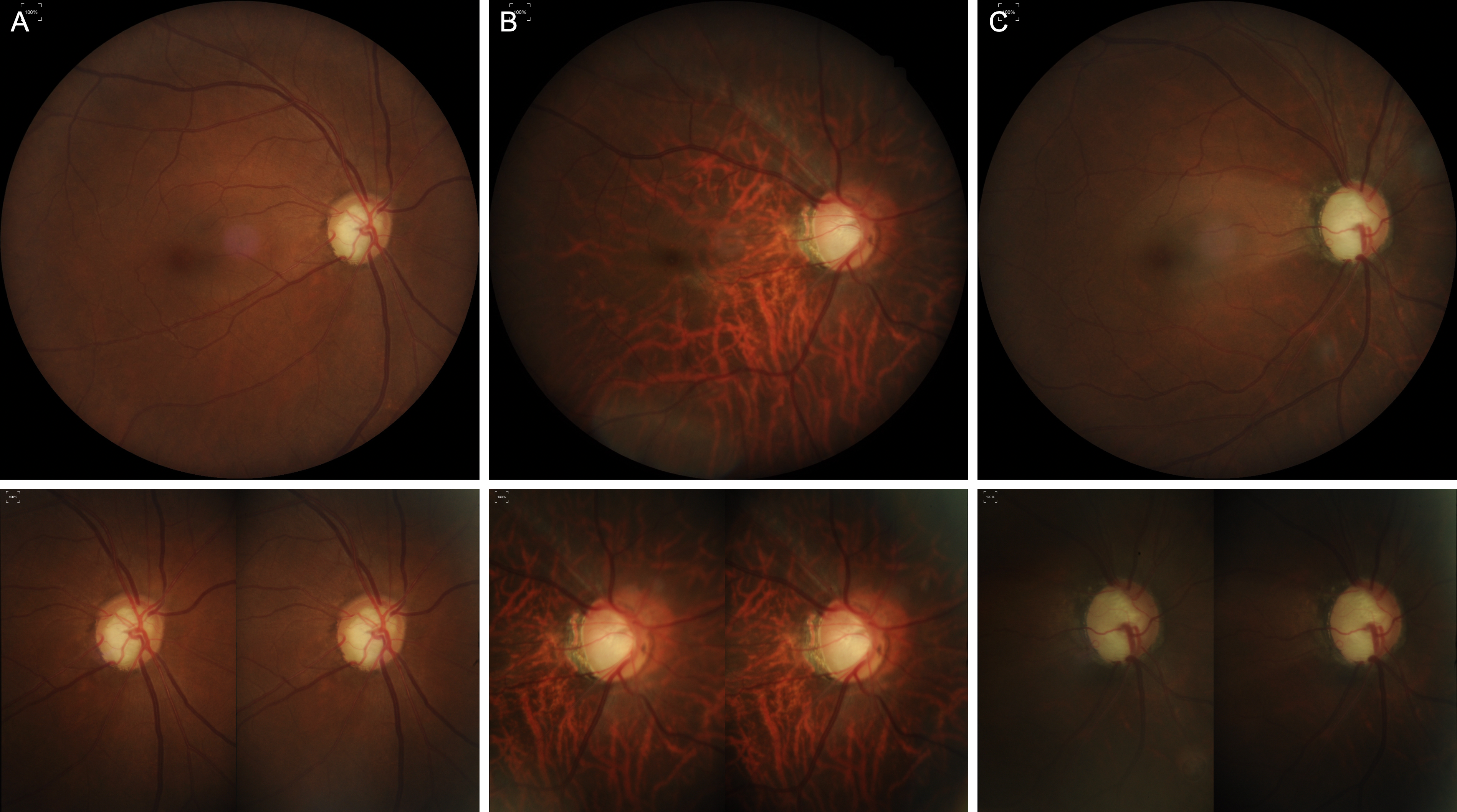

Supplement: Supplementary Figure 1 — Examples of stereoscopic and monoscopic photographs from three glaucomatous eyes. A pair of suitable stereo glasses were strongly suggested when viewing the stereoscopic photographs. [file Image_1.PNG]
