# Supplementary material for: Stereoscopic vs. monoscopic photographs on optic disc evaluation and glaucoma diagnosis among general ophthalmologists: A cloud-based real-world multicenter study
Source: Front Med (Lausanne). 2022 Oct 13;9:990611. doi: 10.3389/fmed.2022.990611 (PMC9612717; doi:10.3389/fmed.2022.990611)
Supplement: Supplementary Table 1 — The number of pairs of stereoscopic and monoscopic photographs evaluated by graders. [file Data_Sheet_1.PDF]

Table S1. The number of pairs of stereoscopic and monoscopic photographs evaluated by graders

No. pairs of photographs	No. graders
257	1
260	1
265	1
288	1
297	1
299	2
300	14

Table S2. The levels of agreement for disc size

Glaucoma likelihood	Viewing method	Agreement between estimates and ground truth		Inter-grader agreement		Agreement between two viewing methods	
		Kappa	95% CI	Kappa	95% CI	Kappa	95% CI
Overall	Stereoscopic	0.447*	0.356-0.539	0.347*	0.323-0.370	0.252	0.137-0.367
	Monoscopic	0.183*	0.121-0.244	0.276*	0.256-0.296		
Definite	Stereoscopic	0.472*	0.391-0.553	0.410*	0.384-0.436	0.304	0.175-0.434
	Monoscopic	0.191*	0.107-0.275	0.255*	0.209-0.301		
Probable	Stereoscopic	0.302*	0.123-0.481	0.398*	0.336-0.461	0.529	0.279-0.780
	Monoscopic	0.137*	0.050-0.224	0.537*	0.452-0.622		
Suspect	Stereoscopic	0.500*	0.379-0.621	0.345*	0.308-0.381	0.182	0.078-0.286
	Monoscopic	0.196*	0.121-0.271	0.160*	0.120-0.200		
None	Stereoscopic	0.353*	0.233-0.473	0.249*	0.219-0.278	0.220	0.080-0.359

	Monoscopic	0.133*	0.061-0.205	0.085*	0.056-0.114		

CI: confidence interval.

* Statistically significant.

Table S3. The levels of agreement for disc color

Glaucoma likelihood	Viewing method	Agreement with ground truth		Inter-grader agreement		Agreement between two viewing methods	
		Kappa	95% CI	Kappa	95% CI	Kappa	95% CI
Overall	Stereoscopic	0.612*	0.565-0.659	0.531*	0.516-0.546	0.619	0.592-0.646
	Monoscopic	0.549*	0.490-0.608	0.505*	0.492-0.519		
Definite	Stereoscopic	0.694	0.643-0.745	0.653*	0.630-0.676	0.701	0.643-0.760
	Monoscopic	0.622	0.544-0.700	0.540*	0.507-0.573		
Probable	Stereoscopic	0.290	0.044-0.537	0.387*	0.314-0.461	0.824	0.621-1
	Monoscopic	0.235	0.010-0.460	0.713*	0.636-0.790		
Suspect	Stereoscopic	0.508*	0.448-0.569	0.410*	0.389-0.432	0.475	0.382-0.568
	Monoscopic	0.392*	0.326-0.459	0.367*	0.336-0.398		
None	Stereoscopic	0.589	0.511-0.667	0.505*	0.483-0.527	0.632	0.583-0.681
	Monoscopic	0.552	0.473-0.631	0.406*	0.369-0.444		

CI: confidence interval.

* Statistically significant.

Table S4. The levels of agreement for disc shape

Glaucoma likelihood	Viewing method	Agreement with ground truth		Inter-grader agreement		Agreement between two viewing methods	
		Kappa	95% CI	Kappa	95% CI	Kappa	95% CI
Overall	Stereoscopic	0.409	0.334-0.483	0.355	0.327-0.383	0.568	0.485-0.652
	Monoscopic	0.339	0.259-0.419	0.344	0.322-0.365		
Definite	Stereoscopic	0.341	0.262-0.420	0.271*	0.238-0.304	0.480	0.367-0.593
	Monoscopic	0.264	0.153-0.376	0.226*	0.184-0.267		
Probable	Stereoscopic	0.286	0.075-0.496	0.505	0.437-0.573	0.882	0.712-1.000
	Monoscopic	0.353	0.1-0.606	0.515	0.430-0.600		
Suspect	Stereoscopic	0.334	0.241-0.427	0.295*	0.258-0.332	0.421	0.268-0.574
	Monoscopic	0.289	0.210-0.369	0.245*	0.207-0.283		
None	Stereoscopic	0.535	0.444-0.627	0.479	0.444-0.514	0.718	0.611-0.825
	Monoscopic	0.468	0.380-0.556	0.470	0.426-0.513		

CI: confidence interval.

* Statistically significant.

Table S5. The levels of agreement for disc contour

Glaucoma likelihood	Viewing method	Agreement with ground truth		Inter-grader agreement		Agreement between two viewing methods	
		Kappa	95% CI	Kappa	95% CI	Kappa	95% CI
Overall	Stereoscopic	0.063	-0.013-	0.172	0.125-	0.278	0.081-

			0.139		0.219		0.474
	Monoscopic	0.088	-0.025-0.202	0.144	0.110-0.179		
Definite	Stereoscopic	0.714	0.504-0.925	0.532	0.465-0.599	0.672	0.431-0.913
	Monoscopic	0.765	0.540-0.990	0.570	0.485-0.655		
Probable	Stereoscopic	1	1	1	1	1	1
	Monoscopic	1	1	1	1		
Suspect	Stereoscopic	0.524	0.291-0.757	0.326	0.264-0.387	0.503	0.259-0.748
	Monoscopic	0.588	0.327-0.849	0.365	0.285-0.444		
None	Stereoscopic	0.121	-0.019-0.260	0.377	0.312-0.442	0.554	0.317-0.791
	Monoscopic	0.102	-0.011-0.216	0.356	0.276-0.436		

CI: confidence interval.

Table S6. The levels of agreement for neuroretinal rim shape

Glaucoma likelihood	Viewing method	Agreement with ground truth		Inter-grader agreement		Agreement between two viewing methods	
		Kappa	95% CI	Kappa	95% CI	Kappa	95% CI
Overall	Stereoscopic	0.356*	0.287-0.426	0.322*	0.300-0.344	0.487	0.375-0.598
	Monoscopic	0.274*	0.199-0.350	0.286*	0.268-0.305		
Definite	Stereoscopic	0.190	0.007-0.374	0.190*	0.148-0.232	0.386	0.225-0.546
	Monoscopic	0.118	-0.053-0.288	0.108*	0.068-0.148		
Probable	Stereoscopic	0.429	0.198-0.659	0.486*	0.418-0.554	0.765	0.540-0.990

	Monoscopic	0.412	0.151-0.673	0.368*	0.286-0.450		
Suspect	Stereoscopic	-0.050	-0.103-0.004	0.144	0.116-0.171	0.319	0.207-0.431
	Monoscopic	-0.019	-0.083-0.046	0.126	0.093-0.158		
None	Stereoscopic	0.248	0.165-0.331	0.182	0.154-0.211	0.502	0.375-0.630
	Monoscopic	0.234	0.148-0.319	0.165	0.127-0.202		

CI: confidence interval.

* Statistically significant.

Table S7. The levels of agreement for ISNT rule consistency

Glaucoma likelihood	Viewing method	Agreement with ground truth		Inter-grader agreement		Agreement between two viewing methods	
		Kappa	95% CI	Kappa	95% CI	Kappa	95% CI
Overall	Stereoscopic	0.353	0.287-0.418	0.255	0.234-0.277	0.427	0.322-0.533
	Monoscopic	0.286	0.205-0.368	0.242	0.224-0.259		
Definite	Stereoscopic	0.251	0.094-0.408	0.133	0.099-0.168	0.311	0.155-0.467
	Monoscopic	0.197	0.052-0.341	0.141	0.102-0.180		
Probable	Stereoscopic	0.476	0.243-0.709	0.390*	0.324-0.457	0.588	0.327-0.849
	Monoscopic	0.412	0.151-0.673	0.301*	0.223-0.380		
Suspect	Stereoscopic	0.003	-0.04-0.046	0.083*	0.056-0.110	0.339	0.236-0.443
	Monoscopic	-0.002	-0.039-0.036	0.143*	0.114-0.172		

None	Stereoscopic	0.220	0.145-0.294	0.153	0.125-0.180	0.371	0.217-0.524
	Monoscopic	0.282	0.186-0.378	0.170	0.133-0.208		

CI: confidence interval; ISNT: I for inferior, S for superior, N for nasal, T for temporal.

* Statistically significant.

Table S8. The levels of agreement for CDR and comparison of different viewing methods

Glaucoma likelihood	Viewing method	Mean	Standard deviation	*P*	Inter-grader agreement	
					ICC	95% CI
Vertical CDR						
Overall	Stereoscopic	0.690	0.111	0.003	0.807	0.787-0.825
	Monoscopic	0.684	0.106			
Definite	Stereoscopic	0.736	0.106	0.056	0.817	0.783-0.846
	Monoscopic	0.730	0.102			
Probable	Stereoscopic	0.657	0.085	0.411	0.782	0.605-0.754
	Monoscopic	0.649	0.070			
Suspect	Stereoscopic	0.686	0.101	0.012	0.735	0.689-0.775
	Monoscopic	0.677	0.099			
None	Stereoscopic	0.647	0.111	0.717	0.800	0.761-0.833
	Monoscopic	0.645	0.101			
Area CDR						
Overall	Stereoscopic	0.449	0.133	0.001	0.856	0.841-0.870
	Monoscopic	0.443	0.125			
Definite	Stereoscopic	0.494	0.138	0.001	0.879	0.855-0.899
	Monoscopic	0.483	0.132			
Probable	Stereoscopic	0.404	0.098	0.051	0.791	0.620-0.890
	Monoscopic	0.425	0.089			
Suspect	Stereoscopic	0.432	0.123	0.031	0.819	0.786-0.847
	Monoscopic	0.424	0.116			
None	Stereoscopic	0.424	0.131	0.481	0.843	0.812-0.870
	Monoscopic	0.422	0.118			

CDR: cup-to-disc ratio; CI: confidence interval; ICC: intraclass correlation coefficient.

Table S9. The levels of agreement for RNFLD

Glaucoma likelihood	Viewing method	Agreement with ground truth		Inter-grader agreement		Agreement between two viewing methods	
		Kappa	95% CI	Kappa	95% CI	Kappa	95% CI
Overall	Stereoscopic	0.362	0.293-0.432	0.293	0.270-0.317	0.538	0.444-0.633
	Monoscopic	0.340	0.244-0.437	0.282	0.262-0.302		
Definite	Stereoscopic	0.190	0.007-0.374	0.091*	0.054-0.129	0.429	0.235-0.623
	Monoscopic	0.176	-0.026-0.379	0.165*	0.116-0.214		
Probable	Stereoscopic	0.333	0.113-0.553	0.379*	0.313-0.446	0.373	0.109-0.636
	Monoscopic	0.176	-0.026-0.379	0.488*	0.411-0.564		
Suspect	Stereoscopic	0.170	0.068-0.272	0.221	0.191-0.250	0.441	0.357-0.525
	Monoscopic	0.115	0.009-0.220	0.181	0.139-0.222		
None	Stereoscopic	0.174	0.059-0.289	0.160	0.113-0.206	0.569	0.438-0.699
	Monoscopic	0.225	0.099-0.352	0.184	0.135-0.234		

CI: confidence interval; RNFLD: retinal nerve fiber layer defect.

* Statistically significant.

Table S10. The levels of agreement for beta zone

Glaucoma likelihood	Viewing method	Agreement with ground truth	Inter-grader agreement	Agreement between two viewing methods

		Kappa	95% CI	Kappa	95% CI	Kappa	95% CI
Overall	Stereoscopic	0.532	0.432-0.632	0.431	0.401-0.461	0.623	0.501-0.744
	Monoscopic	0.568	0.458-0.677	0.441	0.418-0.465		
Definite	Stereoscopic	0.479	0.346-0.611	0.419*	0.378-0.460	0.632	0.503-0.760
	Monoscopic	0.589	0.448-0.730	0.496*	0.451-0.540		
Probable	Stereoscopic	0.619	0.393-0.846	0.395	0.323-0.467	0.471	0.206-0.735
	Monoscopic	0.588	0.327-0.849	0.422	0.339-0.504		
Suspect	Stereoscopic	0.452	0.340-0.564	0.329*	0.298-0.360	0.565	0.430-0.700
	Monoscopic	0.538	0.413-0.663	0.385*	0.340-0.430		
None	Stereoscopic	0.559	0.457-0.662	0.494	0.460-0.528	0.646	0.504-0.789
	Monoscopic	0.536	0.430-0.642	0.474	0.433-0.515		

CI: confidence interval.

* Statistically significant.

Table S11. The levels of agreement for contour of beta zone

Glaucoma likelihood	Viewing method	Agreement with ground truth		Inter-grader agreement		Agreement between two viewing methods	
		Kappa	95% CI	Kappa	95% CI	Kappa	95% CI
Overall	Stereoscopic	-0.005	-0.055-0.044	0.234	0.176-0.291	0.455	0.243-0.667
	Monoscopic	0.109	-0.027-0.246	0.214	0.170-0.257		
Definite	Stereoscopic	0.619	0.393-	0.488*	0.421-	0.627	0.402-

			0.846		0.555		0.852
	Monoscopic	0.529	0.265-0.794	0.287*	0.209-0.366		
Probable	Stereoscopic	0.778	0.565-0.991	0.631	0.552-0.709	0.600	0.319-0.881
	Monoscopic	0.733	0.480-0.987	0.574	0.476-0.672		
Suspect	Stereoscopic	-0.033	-0.091-0.025	0.332*	0.254-0.410	0.611	0.375-0.847
	Monoscopic	0.028	-0.059-0.115	0.201*	0.119-0.284		
None	Stereoscopic	0.026	-0.068-0.119	0.416*	0.334-0.499	0.449	0.182-0.716
	Monoscopic	0.122	-0.070-0.315	0.222*	0.134-0.309		

CI: confidence interval.

* Statistically significant.

Table S12. The levels of agreement for retinal and optic disc hemorrhages

Glaucoma likelihood	Viewing method	Agreement with ground truth		Inter-grader agreement		Agreement between two viewing methods	
		Kappa	95% CI	Kappa	95% CI	Kappa	95% CI
Overall	Stereoscopic	0.476	0.243-0.709	0.339	0.277-0.402	0.597	0.353-0.842
	Monoscopic	0.412	0.151-0.673	0.329	0.282-0.377		
Definite	Stereoscopic	0.905	0.768-1.000	0.847	0.798-0.897	0.882	0.712-1.000
	Monoscopic	0.882	0.712-1.000	0.784	0.715-0.854		
Probable	Stereoscopic	1	1	1	1	1	1
	Monoscopic	1	1	1	1		
Suspect	Stereoscopic	0.571	0.341-	0.456	0.388-	0.765	0.540-

			0.802		0.524		0.990
	Monoscopic	0.529	0.265-0.794	0.504	0.421-0.587		
None	Stereoscopic	0.81	0.626-0.993	0.695	0.632-0.758	0.882	0.712-1.000
	Monoscopic	0.882	0.712-1.000	0.779	0.709-0.850		

CI: confidence interval.

Table S13. The levels of agreement for small vessels

Glaucoma likelihood	Viewing method	Agreement with ground truth		Inter-grader agreement		Agreement between two viewing methods	
		Kappa	95% CI	Kappa	95% CI	Kappa	95% CI
Overall	Stereoscopic	0.239	0.170-0.308	0.163	0.143-0.183	0.403	0.282-0.524
	Monoscopic	0.232	0.146-0.319	0.154	0.136-0.171		
Definite	Stereoscopic	0.152	0.069-0.235	0.131	0.108-0.153	0.336	0.194-0.477
	Monoscopic	0.118	0.050-0.186	0.125	0.084-0.167		
Probable	Stereoscopic	0.095	-0.042-0.232	0.400	0.333-0.467	0.647	0.394-0.900
	Monoscopic	0.176	-0.026-0.379	0.397	0.314-0.480		
Suspect	Stereoscopic	0.143	0.071-0.216	0.115	0.083-0.146	0.375	0.230-0.519
	Monoscopic	0.178	0.083-0.272	0.094	0.060-0.128		
None	Stereoscopic	0.058	-0.030-0.147	0.147*	0.106-0.188	0.382	0.191-0.574
	Monoscopic	0.093	0.017-0.170	0.228*	0.165-0.291		

CI: confidence interval.

* Statistically significant.

Table S14. Inter-grader agreement and accuracy of each subcategory of glaucoma likelihood

Glaucoma likelihood	Viewing method	Accuracy	95% CI	Inter-grader agreement	95% CI
Definite	Stereoscopic	0.557	0.424-0.690	0.206*	0.175-0.237
	Monoscopic	0.573	0.423-0.723	0.123*	0.091-0.154
Probable	Stereoscopic	0.238*	0.101-0.375	0.355*	0.292-0.417
	Monoscopic	0.118*	0.005-0.230	0.435*	0.359-0.510
Suspect	Stereoscopic	0.271	0.195-0.348	0.172*	0.145-0.198
	Monoscopic	0.270	0.200-0.340	0.213*	0.183-0.245
None	Stereoscopic	0.709	0.615-0.804	0.136	0.107-0.165
	Monoscopic	0.653	0.523-0.782	0.166	0.132-0.200

CI: confidence interval.

* Statistically significant.

Table S15. Previous studies of comparison between monoscopic and stereoscopic optic disc photos

Author	Study Design (No. images)	Stereoscopic capture method	No. graders	Major evaluating metrics	Glaucoma likelihood classification	Major conclusion
Parkin et al.[6]	21 images from a glaucoma clinic	Sequential capture	2 experts	Cup-to-disc ratio (CDR).	NA	Differences of CDR was noticed between stereoscopic

						and monoscopic methods.
Chan et al.[7]	40 images from a tertiary clinic	Simultaneous capture	14 consultants and 2 fellows	CDR, disc size, disc shape, disc tilt, peripapillary atrophy, cup shape, cup depth, retinal nerve fiber layer loss, and hemorrhage	Certain, probable, possible, and unlikely.	For expert graders, monoscopic method was comparable to stereoscopic method.
Lichter et al.[8]	20 pairs of images	Sequential capture	6 – 16 experts	CDR, and vessel detail and pallor pattern.	NA	Stereoscopic method was more accurate than monoscopic method to identify pathologic optic discs.
Morgan et al.[9]	35 selected images.	Sequential capture	3 trained and experienced graders	CDR	NA	Stereoscopic method had higher CDR estimates and higher interobserver agreement than monoscopic method did.
Varma et al.[10]	75 images from Wills Eye Hospital	Simultaneous capture	6 experts	CDR	Glaucomatous, non-glaucomatous	Stereoscopic method had performance on evaluating CDR. Stereoscopic and monoscopic methods had similar agreements in

	determining a glaucomatous disc.
